# The modified glasgow prognostic score is an independent prognostic indicator in neoadjuvantly treated adenocarcinoma of the esophagogastric junction

**DOI:** 10.18632/oncotarget.24087

**Published:** 2018-01-08

**Authors:** Gerd Jomrich, Marlene Hollenstein, Maximilian John, Andreas Baierl, Matthias Paireder, Ivan Kristo, Aysegül Ilhan-Mutlu, Reza Asari, Matthias Preusser, Sebastian F. Schoppmann

**Affiliations:** ^1^ Department of Surgery, Medical University of Vienna, and Gastroesophageal Tumor Unit, Comprehensive Cancer Center (CCC), 1090 Vienna, Austria; ^2^ Department of Statistics and Operations Research, University of Vienna, 1090 Vienna, Austria; ^3^ Department of Medicine 1, Medical University of Vienna, Comprehensive Cancer Center (CCC), 1090 Vienna, Austria

**Keywords:** adenocarcinoma of the esophagogastric junction, neoadjuvant therapy, mGPS, inflammation, malnutrition

## Abstract

The modified Glasgow Prognostic Score (mGPS) combines the indicators of decreased plasma albumin and elevated CRP. In a number of malignancies, elevated mGPS is associated with poor survival. Aim of this study was to investigate the prognostic role of mGPS in patients with neoadjuvantly treated adenocarcinomas of the esophagogastric junction 256 patients from a prospective database undergoing surgical resection after neoadjuvant treatment between 2003 and 2014 were evaluated. mGPS was scored as 0, 1, or 2 based on CRP (>1.0 mg/dl) and albumin (<35 g/L) from blood samples taken prior (preNT-mGPS) and after (postNT-mGPS) neoadjuvant therapy. Scores were correlated with clinicopathological patients’ characteristics. From 155 Patients, sufficient data was available. Median follow-up was 63.8 months (33.3–89.5 months). In univariate analysis, Cox proportional hazard model shows significant shorter patients OS (*p* = 0.04) and DFS (*p* = 0.02) for increased postNT-mGPS, preNT-hypoalbuminemia (OS: *p* = 0.003; DFS: *p* = 0.002) and post-NT-CRP (OS: *p* = 0.03; DFS: *p* = 0.04). Elevated postNT-mGPS and preNT-hypoalbuminemia remained significant prognostic factors in multivariate analysis for OS (*p* = 0.02; *p* = 0.005,) and DFS (*p* = 0.02, *p* = 0.004) with tumor differentiation and tumor staging as significant covariates. PostNT-mGPS and preNT-hypoalbuminemia are independent prognostic indicators in patients with neoadjuvantly treated adenocarcinomas of the esophagogastric junction and significantly associated with diminished OS and DFS.

## INTRODUCTION

Esophageal Cancer (EC) is the eighth most common cancer worldwide, with less than 20% of patients surviving more than five years. Whereas in western countries the number of esophageal squamous cell carcinoma (ESCC) is declining, the number of adenocarcinomas of the esophagogastric junction (AEG) diagnosed is increasing [1]. Surgical resection in combination with pre-(peri)operative chemo-(radio) therapy has become the current standard regimen for locally advanced AEG [2, 3]. However, despite improvements in surgical techniques and the introduction of modern multimodal therapeutically regimens, survival is still poor for most patients with AEG. 5-year overall survival (OS) rates after surgery in cases of neoadjuvantly treated adenocarcinomas of the esophagogastric junction (nAEG) reportedly range from 23 to 38% [4]. After surgical resection of nAEG, prognosis has been found to be dependent on traditional tumor-based risk factors, including size, differentiation, lymph node involvement and status of resection margin [5–8]. Most of these factors are determined after surgery only and in addition, these traditional tumor-based risk factors used in clinical practice are influenced by the use of neoadjuvant treatment [9]. Therefore, it is convenient to investigate potential preoperatively available prognostic factors.

In a number of malignancies, tumor associated inflammation, both locally in the tumor microenvironment and as a generalized host response, has been reported to play a prognostic role [10–12], respectively. There is increasing data that the presence of a systemic inflammatory response (SIR) and malnutrition are associated with poor outcomes in patients suffering from advanced cancer stages. Recent studies have revealed that inflammation-based prognostic scores, including the modified Glasgow prognostic score (mGPS), are useful scoring systems for the prognostication in cancer patients [13–19].

Until now, no study evaluated the usefulness of the mGPS in a cohort of patients undergoing esophageal resection for nAEG. Therefore, the aim of this study was to investigate the value of the mGPS for prediction of postoperative overall survival (OS) and disease free survival (DFS) in patients with nAEG.

## RESULTS

A total of 155 neoadjuvantly treated patients undergoing potentially curative gastro-esophageal surgery were included in this study. 144 patients (92.9%) received nCTX and 11 patients (7.1%) received nCRTX. The median follow-up time was 63.8 months (33.3–89.5 months) for OS and 64.1 months (36.1–89.5 months) for DFS. Mean age was 62 (± 10.6 SD) years, and the majority of patients was male (131, 84.5%). There were 105 cases of AEG I, 31 AEG II and 19 AEG III, the most frequent tumor differentiation was yG3 in 84 (54.2%) patients. Most patients (88, 56.8%) showed ypT3 stage and a slight majority of patients (58, 37.4%) showed no nodal involvement after neoadjuvant treatment. In both, pre- and postoperative staging, the majority of cases were present in UICC stage III. 13 patients (8.4%) showed pathological complete regression of the primary tumor (Mandard 1) after surgery. 93 patients were classified as partial responders (Mandard 2–4), whereas 49 (31.6%) patients were categorized as pathological non-responders (Mandard 5-absence of regressive changes). Adjuvant chemotherapy was administered in 44 (28.4%) patients. For Details for patients’ characteristics and clinicopathological results see Table 1. The median OS was 33.6 months (range 13.5 to 105.4 months) and 17.5 months (range 7.1 to 94.0 months) for DFS.

**Table 1:** Clinicopathologic parameters in neoadjuvant treated patients with adenocarcinoma of the gastroesophageal junction

*Factors*	All Patients (*n* = 155)	(%)
**Mean Age (SD)**	62 (10.6)	
**Sex**		
Male	131	(84.5)
Female	24	(15.5)
**cT**		
1	0	(0.0)
2	46	(29.7)
3	105	(67.7)
4	4	(2.6)
**cN**		
0	25	(16.1)
1	99	(63.9)
2	31	(20.0)
**UICC Stage preNT**		
I	17	(11.0)
II	33	(21.3)
III	105	(67.7)
**ypT**		
0	13	(8.4)
1	18	(11.6)
2	28	(18.1)
3	88	(56.8)
4	8	(5.2)
**ypN**		
0	58	(37.4)
1	57	(36.8)
2	18	(11.6)
3	22	(14.2)
**yG**		
0	13	(8.4)
1	1	(0.6)
2	56	(36.1)
3	84	(54.2)
4	1	(0.6)
**UICC Stage postNT**		
0	10	(6.5)
I	23	(14.8)
II	40	(25.8)
III	82	(52.9)
**Mandard Regression**		
1	13	(8.4)
2-4	93	(60.0)
5	49	(31.6)
**AEG**		
I	105	(67.7)
II	31	(20.0)
III	19	(12.3)
**Adjuvant Therapy**		
yes	44	(28.4)
no	111	(71.6)

SD = standard deviation; UICC = Union for International Cancer Control.

AEG = adenocarcinoma of the esophagogastric junction; CRP = C-reactive protein.

### Pre-Neoadjuvant Therapy (preNT) mGPS

**Figure 1:**
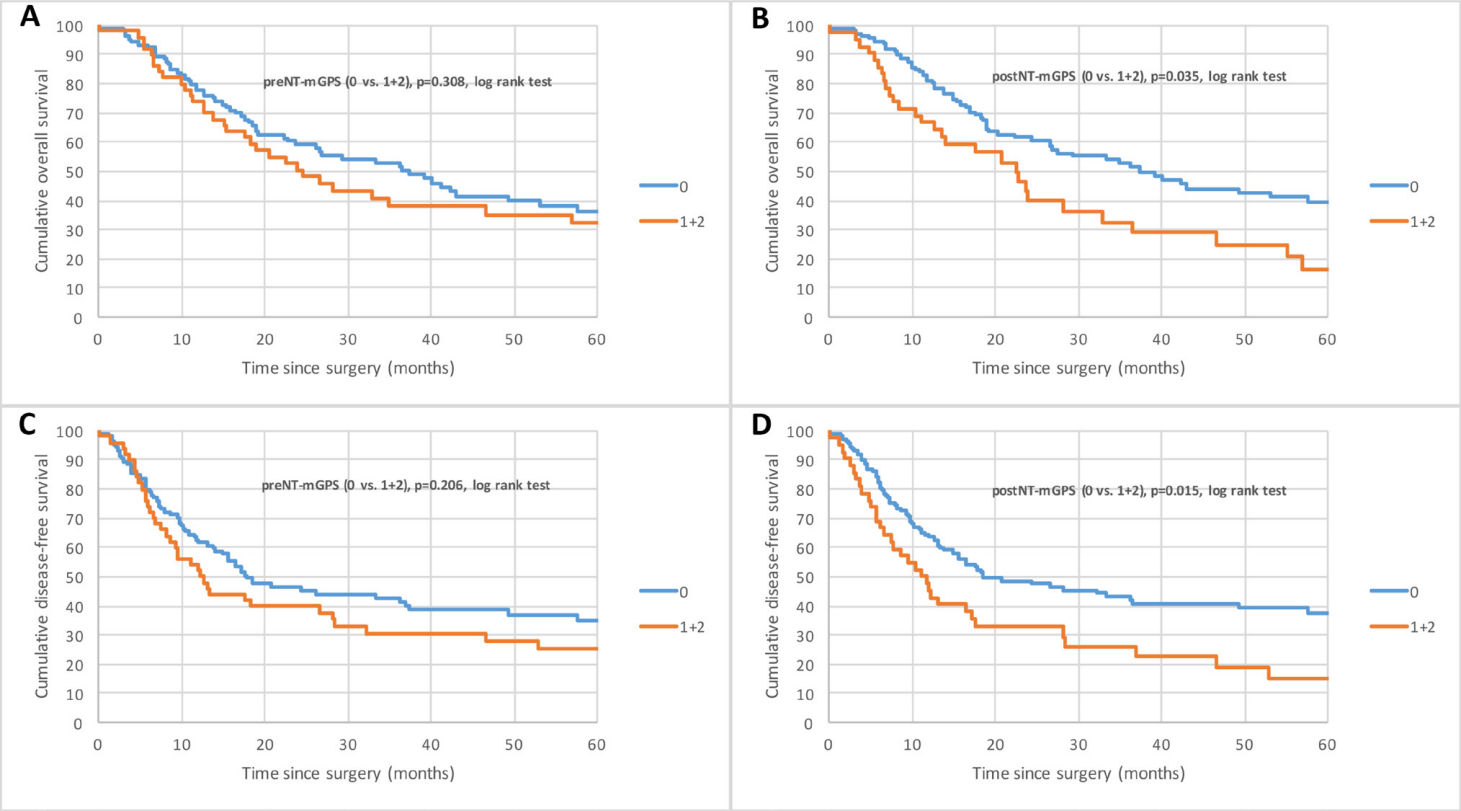
Kaplan–Meier curves for survival of nAEG patients with mGPS 0 compared with mGPS 1 and 2 (**A** and **B**) Overall survival (OS) for preNT-mGPS and postNT mGPS. (**C** and **D**) Disease-free survival (DFS) for preNT-mGPS and postNT-mGPS.

Analyzing the preNT laboratory results, 73 (47.1%) patients showed elevated plasma CRP with a mean preNT CRP level of 1.6 mg/dl (± 3.1). PreNT-hypoalbuminemia was found in 29 (18.7%) patients (mean 39.60 g/l (± 6.60)) (Table 2). 105 (67.7%) patients had a preNT-mGPS of 0 and 50 patients (32.3%) a preNT-mGPS of 1 or 2. Patients with elevated preNT-mGPS were more likely to be male (43, 86.0%), showed a higher tumor grading (29, 58.0%) and a higher ypT staging (32, 64.0%) at the time of surgery (Supplementary Table 1). Univariate Cox proportional hazard regression showed that preNT-hypoalbuminemia was associated with poor OS and DFS (*p* = 0.003, *p* = 0.002). No significant correlation was found for the patients’ groups preNT-mGPS 0 vs. 1 and 2 and OS (*p* = 0.308) and DFS (*p* = 0.206) (Figure 1A and 1C).

**Table 2:** Values of serum albumin, CRP and mGPS before and after neoadjuvant treatment

*Factors*	All Patients (*n* = 155)	(%)
**preNT-Mean Albumin (SD)**	39,6 (6,6)	
<35.0 g/L	29	(18.7)
≥35.0 g/L	126	(81.3)
**preNT - Mean CRP (SD)**	1,6 (3,1)	
<0.5 mg/dL	82	(52.9)
≥0.5 mg/dL	73	(47.1)
**preNT - mGPS**		
0	105	(67.7)
1 + 2	50	(32.3)
**postNT - Mean Albumin (SD)**	39,5 (6,1)	
<35.0 g/L	28	(18.1)
≥35.0 g/L	127	(81.9)
**postNT - Mean CRP (SD) -**	1,88 (1,1)	
<0.5 mg/dL	89	(57.4)
≥0.5 mg/dL	66	(42.6)
**postNT - mGPS**		
0	113	(72.9)
1+2	42	(27.1)

mGPS = modified Glasgow Prognostic Score; preNT = prior to neoadjuvant therapy.

postNT = after neoadjuvant therapy; SD = standard deviation; CRP = C-reactive protein.

Based on univariate Cox regression, hypoalbuminemia and tumor differentiation were identified as significant prognostic factors for OS, and hypoalbuminemia, sex, tumor differentiation and UICC stage for DFS (Supplementary Table 3). Stepwise regression analysis for multivariate Cox models identified preNT-hypoalbuminemia (*p* = 0.005, RR 0.52, CI95% 0.33–0.82) and tumor differentiation (*p* = 0.001, RR 0.49, CI95% 0.31–0.75) as independent risk factors for OS, and preNT-hypoalbuminemia (*p* = 0.004, RR 0.51, CI95% 0.33–0.80) and UICC stage (*p* = 0.006, RR 0.57, CI95% 0.38–0.85) for DFS (Table 3).

**Table 3:** Multivariate Cox regression analysis estimating the influence of mGPS and clinicopathological parameters on overall survival and disease free survival

preNT	*p* value multivariate	HR	95% CI
*Overall Survival (OS)*			
**preNT - Albumin (≥35.0 g/l vs. <35.0 g/l)**	0.005	0.52	0.33–0.82
**yG (G0, G1 and G2 vs. G3 and G4)**	0.001	0.49	0.31–0.75
*Disease Free Survival (DFS)*			
**preNT - Albumin (≥35.0 g/l vs. <35.0 g/l)**	0.004	0.51	0.33–0.80
**UICC Stage (I and II vs. III)**	0.006	0.57	0.38–0.85
**postNT**			
*Overall Survival (OS)*			
**mGPS (0 vs. 1+2)**	0.017	1.72	1.10–2.67
**yG (G0, G1 and G2 vs. G3 and G4)**	<0.001	0.46	0.30–0.71
*Disease Free Survival (DFS)*			
**mGPS (0 vs. 1+2)**	0.0195	1.65	1.08–2.50
**yG (G0, G1 and G2 vs. G3 and G4)**	0.0145	0.60	0.39–0.90
**UICC Stage (0, I, II vs. III)**	0.0263	0.63	0.42–0.95

mGPS = modified Glasgow Prognostic Score; preNT = prior to neoadjuvant therapy; postNT = after neoadjuvant therapy;

UICC = Union for International Cancer Control; CI = confidence interval; CRP = C-reactive protein.

### Post-Neoadjuvant Therapy (postNT) mGPS

Elevated postNT-CRP levels were found in 66 patients (42.6%). The mean postNT CRP level was 1.88 mg/dl (±1.10). Decreased serum albumin was measured in 28 patients (18.1%). The mean postNT albumin level was 39.50 g/l (±6.10) (Table 2). 113 (72.9%) patients had a postNT-mGPS 0 and 42 (27.1%) a postNT-mGPS 1 or 2. Patients with elevated postNT-mGPS were more likely to be male (37, 88.1%), had a high tumor differentiation (24, 57.1%) and a larger tumor (26, 61.9%) at the time of surgery (Supplementary Table 2). Kaplan-Meier survival analysis and univariate Cox proportional hazard regression demonstrated that postNT-mGPS is associated with diminished OS (*p* = 0.04) and DFS (*p* = 0.02) in neoadjuvantly treated AEG patients (Figure 1B and 1D; Supplementary Table 4).

It was found, that postNT-CRP, postNT-albumin and postNT-mGPS were highly correlated due to the same factors. Stepwise regression analysis for multivariate Cox models revealed that postNT-mGPS (*p* = 0.017, RR 1.72, CI95% 1.10–2.67) and tumor differentiation (*p* < 0.001, RR 0.46, CI95% 0.30–0.71), are independent risk factors for OS, postNT-mGPS (*p* = 0.0195, RR 1.65, CI95% 1.08–2.50), tumor differentiation (*p* = 0.0145, RR 0.60, CI95% 0.39–0.90) and UICC (*p* = 0.0263, RR 0.63, CI95% 0.42–0.95) for DFS in our cohort (Table 3).

## DISCUSSION

To the best of our knowledge, this is the first report to demonstrate the prognostic role of mGPS in patients with neoadjuvantly treated AEG. In this study, we revealed that elevated preNT-hypoalbuminemia and postNT-mGPS are highly associated with significantly impaired survival in patients suffering from neoadjuvantly treated AEG.

In 1863, Rudolf Virchow first described the correlation between inflammation and tumor, as a wound that never heals [20]. Inflammation is known to be one of the hallmarks of cancer [21]. Over the last few years, accumulating data verified that inflammation contributes to tumorigenesis, progression and metastasis. In a number of cancers, including EC, inflammation based prognostic scores have been found to be independent prognostic markers [13–19, 22, 23].

The mGPS combines albumin and CRP into a prognostic risk stratification score for predicting the clinical outcome in cancer patients. Even though malnutrition is associated with malignant diseases, the role of hypoalbuminemia in cancer patients has to be seen critical. Recently, we could show that sarcopenia, another marker for malnutrition, impacts long-term outcome after esophageal resection in patients who have undergone neoadjuvant therapy [24]. In 2008, McMillan *et al.* reported that hypoalbuminemia alone reflect a systemic inflammatory response [25]. On the other hand, the same study group could recently show, that CRP, but not albumin is an independent prognostic factor in gastric cancer patients [26]. This data goes in good accordance with our recently published data, that shows, that albumin and CRP based prognostic scores have to be used carefully in EC patients. Nevertheless, in the subgroup of neoadjuvantly treated AEG patients, we found the mGPS to be a prognostic marker [27]. Therefore, we evaluated the mGPS in a cohort neoadjuvantly treated AEG patients in this underlying study.

In contrast to previously published data, addressing the factor albumin as the weak point in mGPS, we found, that preNT-hypoalbuminemia is an independent prognostic factor. The importance of pre preNT-hypoalbuminemia is corroborated by the fact, that preNT-CRP, and in addition preNT-mGPS were not to be found as prognostic factors.

Recently, Feng *et al.* showed, that the systemic immune-inflammation index (SII) is an independent prognostic indicator for patients with resectable ESCC without neoadjuvant treatment. Further, this study group hypothesizes, that the investigation of an inflammation based prognostic score in neoadjuvantly treated EC patients, has to be seen critical, due to the fact, that neoadjuvant therapy will influence inflammation [22]. In good accordance to this hypothesis, we could show first in this study, that there are differences in the prognostic role of mGPS in nAEG before or after neoadjuvant therapy that have to be considered.

Until now, it remains unclear why the prognostic value of mGPS differs between pre- and post neoadjuvant treatment. One can only hypothesize, that CRP is stronger affected by changes in systemic inflammation induced by neoadjuvant treatment, than albumin. This hypothesis is underpinned by our findings, that albumin is an independent prognostic factor preNT, whereas CRP is an independent prognostic factor postNT.

Though, this is the first study revealing differences of the mGPS pre- and postNT esophageal adenocarcinomas, that have to be mentioned.

In conclusion, mGPS and its components as single factors are independent prognostic factor for patients with nAEG undergoing radical esophagostomy. Nevertheless, there are differences of the prognostic value of preNT-mGPS or postNT-mGPS that have to be mentioned using the mGPS in nAEG patients. In addition, our data shows that preNT- hypoalbuminemia and postNT-mGPS both highly correlate with tumor diffentiation and tumor statging (UICC). This makes the mGPS, based on simple and inexpensive standard laboratory results, a potential marker for nAEG prognosis and treatment response surveillance.

## MATERIALS AND METHODS

### Patients and therapy

Patients who underwent surgical resection for nAEG between 1999 and 2016 at the department of surgery at the Medical University Vienna, were identified from a prospective maintained esophageal cancer database. Patients with histopathological diagnosed and locally advanced adenocarcinoma of the gastroesophageal junction who received radio- and/or chemotherapy and attempted curative resection were included in this study. Exclusion criteria were, distant metastasis at time of surgery, positive resection margin, postoperative death from another cause than cancer, death or postoperative complications, including anastomotic leakage, pneumonia or wound infection within 30 days after surgery, pyrexia before neoadjuvant treatment or surgery (axillary ≥37.2° C / 99.0°F) or any form of active infection or chronic inflammatory disease and missing preoperative levels of albumin and/or C-reactive protein (CRP). None of the patients exhibited clinical evidence of infection or any other inflammatory conditions before neoadjuvant treatment and before surgery. This study was approved by the ethics committee of the Medical University Vienna, Austria, according to the declaration of Helsinki.

Demographic, histopathologic and laboratory variables including serum albumin and CRP levels, before neoadjuvant treatment (preNT) and after neoadjuvant treatment (postNT) were retrospectively reviewed and collected from the local databases and patients’ records.

The tumor stage was determined according to the pathological tumor-node-metastasis (pTNM) classification of the Union for International Cancer Control (UICC), 7th edition. Pre- and postoperatively every patient was discussed in the interdisciplinary tumor board meeting. Before surgery, neoadjuvant chemotherapy (nCTX) was carried out generally by intravenous infusion, either with oxaliplatin/capecitabine-based or cisplatine/5- fluoruracil-based according to current study protocol. Concomitant radiation (nCRTX) was performed, according to the recommendations of the interdisciplinary tumor board based on the regimen published by Van Hagen *et al.* [28].

Response rate to neoadjuvant treatment was classified as defined by Mandard A.M. *et al.* [29].

The tumor location of tumors at the gastroesophageal junction was classified according to Siewert *et al.* [30].

Transhiatal extended gastregectomy (THG) was performed in patients with AEG II and III tumors. Merendino procedure was performed in patients in cases presenting with stage I tumors in AEG I or II located cancers. Abdomino-thoratic esophageal resection (ATE) was performed in patients with AEG I and II tumors.

All patients were regularly followed up with physical examination, tumor marker and computed tomography at our outpatient clinic every 3 month for the first 2 years and every 6 months until 5 years after surgery.

The preNT and postNT serum concentrations of albumin and CRP were measured within 3 days prior to the start of neoadjuvant treatment and surgery, respectively.

CRP levels were determined by particle-enhanced immunoturbidimetry and albumin was quantified by means of colorimetry using bromocresol green (depending on the date of blood testing: Olympus, Tokyo, Japan; Beckman Coulter, Brea, USA; Roche Diagnostics, Rotkreuz, Switzerland) under controlled conditions at the Department of Laboratory Medicine, Medical University of Vienna, which runs as the central laboratory of the General Hospital of Vienna a certified (ISO 9001) and accredited (ISO 15189, since 2008) quality management system [31].

The mGPS was determined as described previously [25, 32, 33]. The plasma CRP and plasma albumin concentration of >1.0 mg/dl and <35.0 g/l were considered pathological. In brief, patients with an elevated CRP (>1.0 mg/dl) level and decreased serum albumin (<35.0 g/l) were assigned a score of 2. Patients with elevated CRP (>1.0 mg/dl) and albumin (≥35.0 g/l) levels were allocated a score of 1 and those with a normal CRP level (≤1.0 mg/dl) were given a score of 0.

Investigating the prognostic role of plasma CRP and plasma albumin individually, concentrations of >0.5 mg/dl and <35.0 g/l were considered as pathological, respectively.

### Statistical analysis

Overall survival (OS) was defined as the time between primary surgery and the patients’ death. Death from cause other than gastroesophageal or esophageal cancer or survival until the end of the observation period was considered as censored observations. Disease free survival (DFS) was defined from the day of primary surgery until the first evidence of disease progression. Data are presented as mean and standard deviation (SD) for continuous variables, as absolute and relative frequency for categorical data, and as median and interquartile range (IQR) for follow up and survival times, respectively. Median follow up was estimated by reverse Kaplan-Meier method. Kaplan-Meier curves were plotted to investigate differences in OS and DFS between mGPS-levels. Univariate Cox proportional hazards models were carried out to estimate the effect of each predictor on OS and DFS, separately. Stepwise regression analysis was applied to select the set of covariates that best predict OS and DFS, respectively, in the setting of a multivariate Cox proportional hazard model. Proportional hazard assumptions were assessed visually and tested using diagnostics based on weighted residuals. All tests were two-sided and p-values less than 0.05 were considered statistically significant. All statistical analyses were performed with the statistical software R version 3.33 [34].

## SUPPLEMENTARY MATERIALS FIGURES AND TABLES







## Abbrevations

AEG: Adenocarcinoma of the esophagogastric junction
CI: Confidence Interval
CRP: C-reactive protein
DFS: Disease-free survival
EC: Esophageal cancer
ESCC: Esophageal squamous cell carcinoma
g/L: Gram per liter
mg/dl: Milligram per deciliter
mGPS: Modified Glasgow Prognostic Score
nCRTX: Neoadjuvant chemo-radio therapy
nCTX: Neoadjuvant chemotherapy
nAEG: Neoadjuvantly treated Adenocarcinoma of the esophagogastric junction
OS: Overall survival
postNT: Post neoadjuvant Therapy
preNT: Pre neoadjuvant Therapy
RR: Relative Risk
SD: Standard deviation
SII: Systemic immune-inflammation index
SIR: Systemic inflammatory response
UICC: The Union for International Cancer Control
