# Supplementary material for: The modified glasgow prognostic score is an independent prognostic indicator in neoadjuvantly treated adenocarcinoma of the esophagogastric junction
Source: Oncotarget. 2018 Jan 8;9(6):6968–76. doi: 10.18632/oncotarget.24087 (PMC5805529; doi:10.18632/oncotarget.24087)
Supplement: Supplementary file 1 [file oncotarget-09-6968-s001.pdf]

The modified glasgow prognostic score is an independent prognostic indicator in neoadjuvantly treated adenocarcinoma of the esophagogastric junction

SUPPLEMENTARY MATERIALS

Supplementary Table 1: Association of mGPS with clinicopathologic parameters before neoadjuvant treatment in AEG patients. See Supplementary_Table_1

Supplementary Table 2: Association of mGPS with clinicopathologic parameters after neoadjuvant treatment in AEG patients. See Supplementary_Table_2

Supplementary Table 3: Univariate Cox regression analysis estimating the influence of mGPS and clinicopathological parameters on overall survival and disease free survival before neoadjuvant treatment

Factors	***p* value univariate**	**HR**	**95% CI**
Overall Survival (OS)			
preNT - CRP (≥0.5 mg/dl vs. <0.5 mg/dl)	0.781	1,06	0,70–1,59
preNT - Albumin (≥35.0 g/l vs. <35.0 g/l)	0.003	0.50	0,32–0,79
preNT - mGPS (0 vs. 1+2)	0.308	1.25	0,82–1,90
Age (±SD)	0.475	0.99	0,98–1,01
Sex	0.095	0.57	0,30–1,10
yG (G0 vs. G3 and G4)	0.064	0.38	0,14–1,06
yG (G1 and G2 vs. G3 and G4)	0.002	0.50	0,31–0,78
UICC Stage (0 vs. III)	0.053	0.32	0,10–1,01
UICC Stage (I and II vs. III)	0.051	0.65	0,43–1,00
Adjuvant Therapy (no vs. yes)	0.854	0.96	0,61–1,50
AEG II vs. I	0.676	1.12	0,67–1,86
AEG III vs. I	0.286	0.68	0,33–1,38
Disease Free Survival (DFS)			
preNT - CRP (≥0.5 mg/dl vs. <0.5 mg/dl)	0.468	1.15	0,78–1,70
preNT - Albumin (≥35.0 g/l vs. <35.0 g/l)	0.002	0.50	0,32–0,78
preNT - mGPS (0 vs. 1+2)	0.206	1.30	0,87–1,93
Age (±SD)	0.216	0.99	0,97–1,01
Sex	0.031	0.49	0,25–0,94
yG (G0 vs. G3 and G4)	0.034	0.33	0,12–0,92
yG (G1 and G2 vs. G3 and G4)	0.018	0.60	0,40–0,92
UICC Stage (0 vs. III)	0.020	0.25	0,08–0,80
UICC Stage (I and II vs. III)	0.010	0.59	0,39–0,88
Adjuvant Therapy (no vs. yes)	0.385	0.83	0,55–1,26
AEG II vs. I	0.907	1.03	0,63–1,69
AEG III vs. I	0.178	0.62	0,31–1,24

mGPS = modified Glasgow Prognostic Score; preNT = prior to neoadjuvant therapy; SD = standard deviation; UICC = Union for International Cancer Control; AEG = adenocarcinoma of the esophagogastric junction; CI = confidence interval; CRP = C-reactive protein.

Supplementary Table 4: Univariate Cox regression analysis estimating the influence of mGPS and clinicopathological parameters on overall survival and disease free survival after neoadjuvant treatment

Factors	***p* value univariate**	**HR**	**95% CI**
Overall Survival (OS)			
postNT - CRP (≥0.5 mg/dl vs. <0.5 mg/dl)	0.027	1.59	1,06–2,40
postNT - Albumin (≥35.0 g/l vs. <35.0 g/l)	0.918	1.02	0,60–1,78
postNT - mGPS (0 vs. 1+2)	0.035	1.61	1,03–2,50
Age (±SD)	0.475	0.99	0,98–1,01
Sex	0.095	0.57	0,30–1,10
yG (G0 vs. G3 and G4)	0.064	0.38	0,14–1,06
yG (G1 and G2 vs. G3 and G4)	0.002	0.50	0,31–0,78
UICC Stage (0 vs. III)	0.053	0.32	0,10–1,01
UICC Stage (I and II vs. III)	0.051	0.65	0,43–1,00
Mandard Response (1-2 vs. 3-5)	0.023	0.45	0,23–0,90
Adjuvant Therapy (no vs. yes)	0.854	0.96	0,61–1,50
AEG II vs. I	0.676	1.12	0,67–1,86
AEG III vs. I	0.286	0.68	0,33–1,38
Disease Free Survival (DFS)			
postNT - CRP (≥0.5 mg/dl vs. <0.5 mg/dl)	0.036	1.52	1,03–2,24
postNT - Albumin (≥35.0 g/l vs. <35.0 g/l)	0.966	1.01	0,61–1,68
postNT - mGPS (0 vs. 1+2)	0.015	1.66	1,10–2,51
Age (±SD)	0.216	0.99	0,97–1,01
Sex	0.031	0.49	0,25–0,94
yG (G0 vs. G3 and G4)	0.034	0.33	0,12–0,92
yG (G1 and G2 vs. G3 and G4)	0.018	0.60	0,40–0,92
UICC Stage (0 vs. III)	0.020	0.25	0,08–0,80
UICC Stage (I and II vs. III)	0.010	0.59	0,39–0,88
Mandard Response (1–2 vs. 3–5)	0.005	0.37	0,19–0,74
Adjuvant Therapy (no vs. yes)	0.385	0.83	0,55–1,26
AEG II vs. I	0.907	1.03	0,63–1,69
AEG III vs. I	0.178	0.62	0,31–1,24

mGPS = modified Glasgow Prognostic Score; postNT = after neoadjuvant therapy; SD = standard deviation; UICC = Union for International Cancer Control; AEG = adenocarcinoma of the esophagogastric junction; CI = confidence interval; CRP = C-reactive protein.
